# Supplementary material for: Single-Disulfide Conopeptide Czon1107, an Allosteric Antagonist of the Human α3β4 Nicotinic Acetylcholine Receptor
Source: Mar Drugs. 2022 Jul 31;20(8):497. doi: 10.3390/md20080497 (PMC9409646; doi:10.3390/md20080497)
Supplement: Supplementary file 1 [file marinedrugs-20-00497-s001.zip › marinedrugs-1825769-SI.pdf]

Single-disulfide Conopeptide Czon1107, an Allosteric Antagonist of the Human α3β4 Nicotinic Acetylcholine Receptor

Yuan Ma [1,†], Qiushi Cao [1,†], Mengke Yang [1,†], Yue Gao [1], Shuiping Fu [1], Wenhao Du [1], David J. Adams [2], Tao Jiang [1,3], Han-Shen Tae [2,*] and Rilei Yu [1,3,4,*]

[1]*Key Laboratory of Marine Drugs, Chinese Ministry of Education, School of Medicine and Pharmacy, Ocean University of China, 5 Yushan Road, Qingdao 266003, China*

[2]*Illawarra Health and Medical Research Institute (IHMRI), University of Wollongong, Wollongong, New South Wales 2522, Australia*

[3]*Laboratory for Marine Drugs and Bioproducts, Qingdao National Laboratory for Marine Science and Technology, Qingdao 266003, China*

[4]*Innovation Center for Marine Drug Screening & Evaluation, Qingdao National Laboratory for Marine Science and Technology, Qingdao 266003, China*

[*] Corresponding authors: hstae@uow.edu.au and ryu@ouc.edu.cn

[†] Authors contributed equally to this manuscript.

Contents

Figure S1. The HPLC of Czon1107 1
Figure S2. The MS of Czon1107 1
Figure S3. The HPLC of Czon1107[G1A] 2
Figure S4. The MS of Czon1107[G1A] 2
Figure S5. The HPLC of Czon1107[F2A] 3
Figure S6. The MS of Czon1107[F2A] 3
Figure S7. The HPLC of Czon1107[R3A] 4
Figure S8. The MS of Czon1107[R3A] 4
Figure S9. The HPLC of Czon1107[S4A] 5
Figure S10. The MS of Czon1107[S4A] 5
Figure S11. The HPLC of Czon1107[P8A] 6
Figure S12. The MS of Czon1107[P8A] 6
Figure S13. The HPLC of Czon1107[F9A] 7
Figure S14. The MS of Czon1107[F9A] 7
Figure S15. The HPLC of Czon1107[G1R] 8
Figure S16. The MS of Czon1107[G1R] 8
Figure S17. The HPLC of Czon1107[F2R] 9
Figure S18. The MS of Czon1107[F2R] 9
Figure S19. The HPLC of Czon1107[S4R] 10
Figure S20. The MS of Czon1107[S4R] 10
Figure S21. The HPLC of Czon1107[P5R] 11
Figure S22. The MS of Czon1107[P5R] 11
Figure S23. The HPLC of Czon1107[P7R] 12
Figure S24. The MS of Czon1107[P7R] 12
Figure S25. The HPLC of Czon1107[P8R] 13
Figure S26. The MS of Czon1107[P8R] 13
Figure S27. The HPLC of Czon1107[F9R 14
Figure S28. The MS of Czon1107[F9R] 14
Figure S29. RMSD of Czon1107 combined with hα3β4 nAChR after MD for 200ns 15
Figure S30. The activity of the labelled Czon1107 analogues on ACh-evoked peak current amplitude mediated by hα3β4 nAChRs. 15
Figure S31. Residue F2 of Czon1107 interacts with hα3β4 and hα7 nAChRs. 16

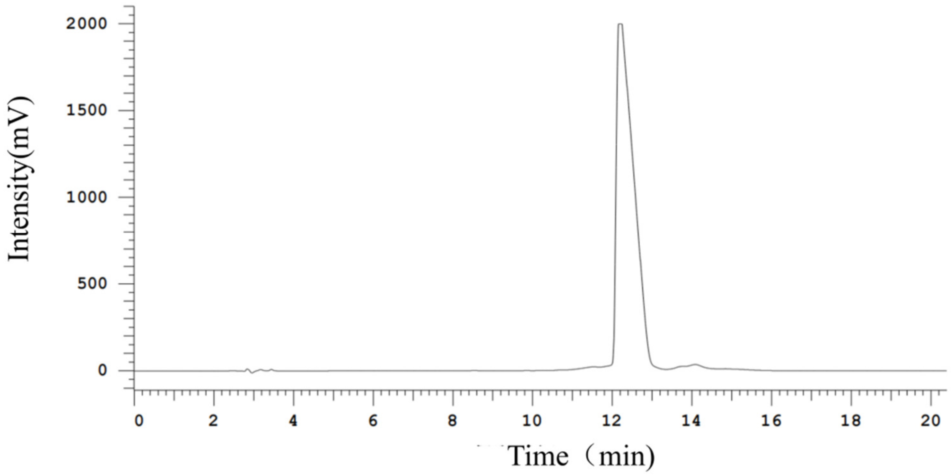

Figure S1. The HPLC of Czon1107

HPLC conditions:

COSMOSIL 5C18-MS-II (4.6mmI.D.x 250mm) was used as the column at 25˚C, and the mobile phase flow rate was 1 mL/min. During the analytical run, the elution was carried out using mobile phases A (90% water, 10% acetonitrile, 0.05% trifuoroacetic acid) and B (90% acetonitrile, 10% water, 0.05% trifuoroacetic acid) of 90% to 60% for 30min with 1% per minute changing of gradient, while the detection wavelength was 220 nm. The following analysis conditions are the same.

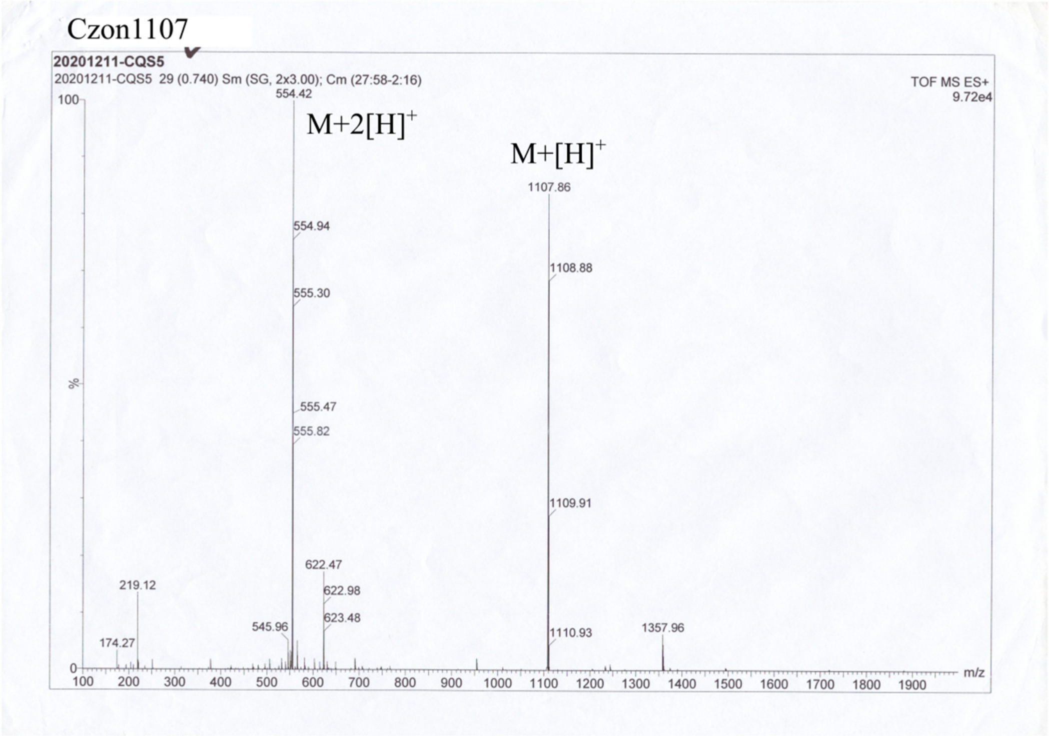

Figure S2. The MS of Czon1107

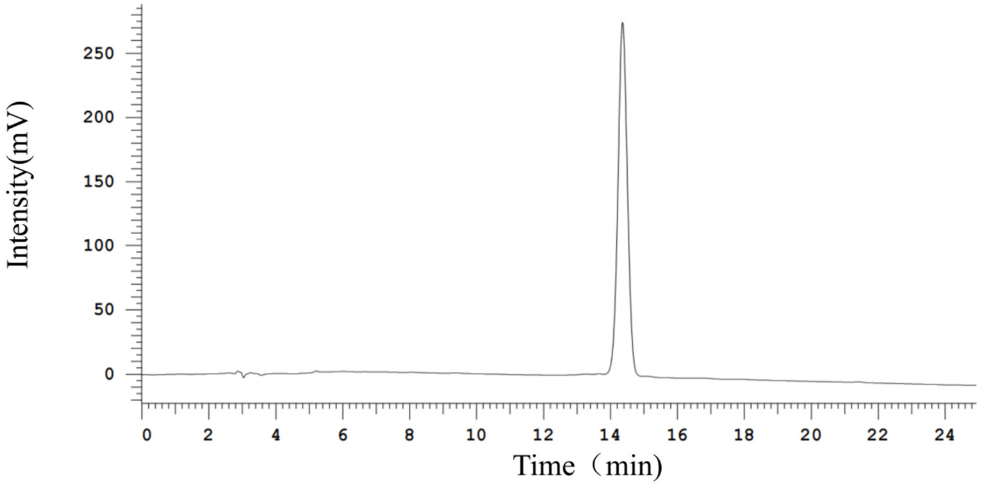

Figure S3. The HPLC of Czon1107[G1A]

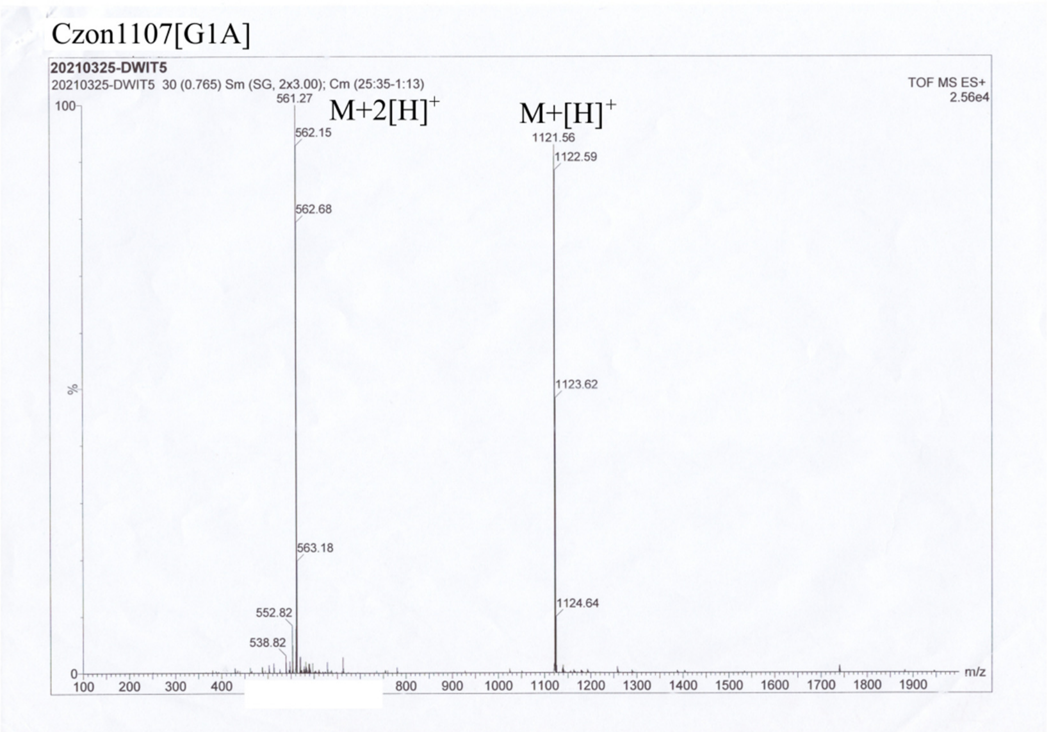

Figure S4. The MS of Czon1107[G1A]

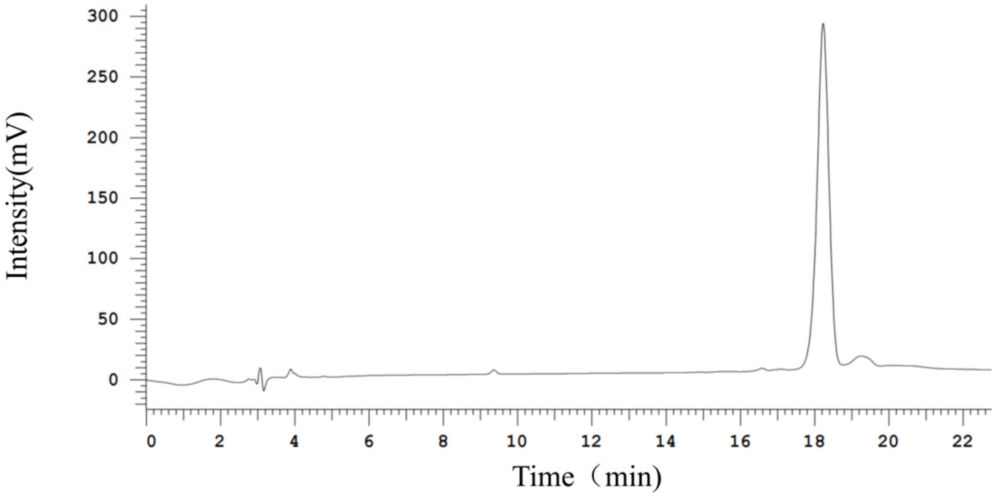

Figure S5. The HPLC of Czon1107[F2A]

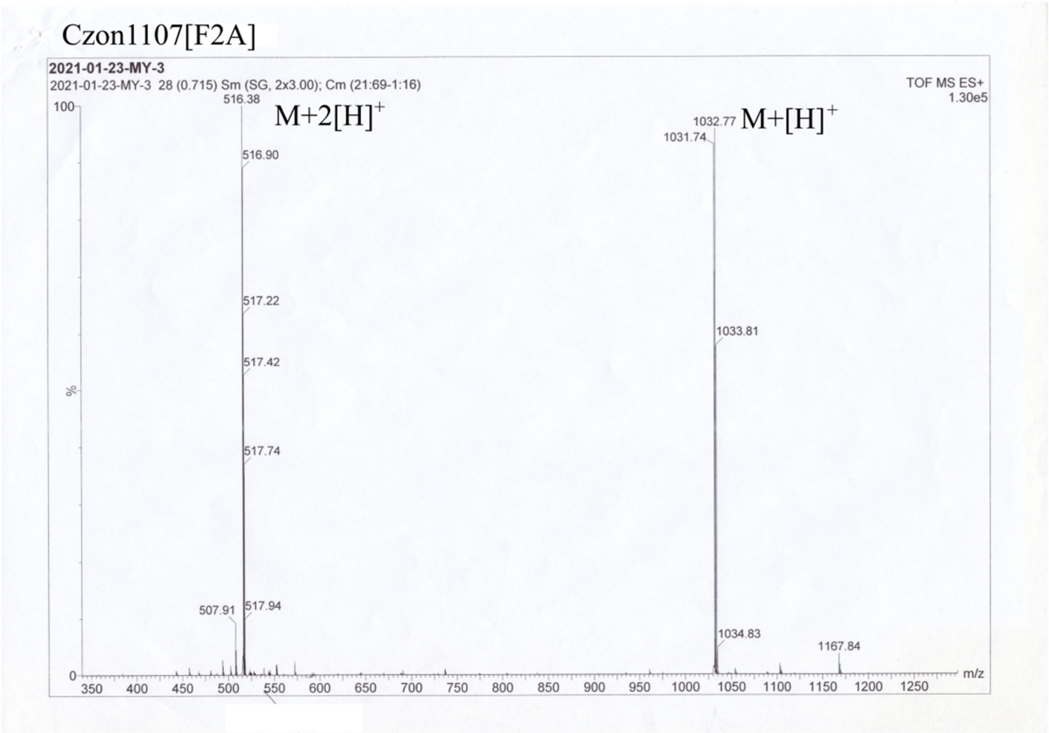

Figure S6. The MS of Czon1107[F2A]

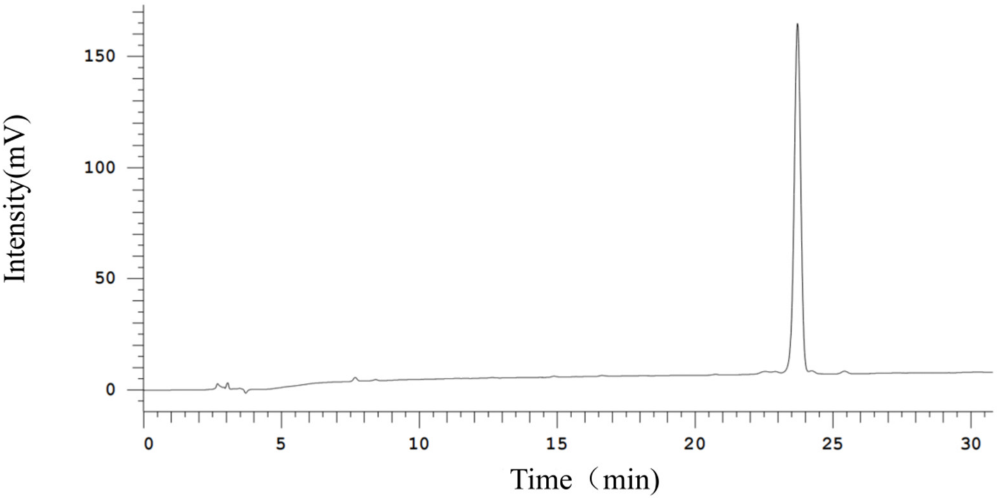

Figure S7. The HPLC of Czon1107[R3A]

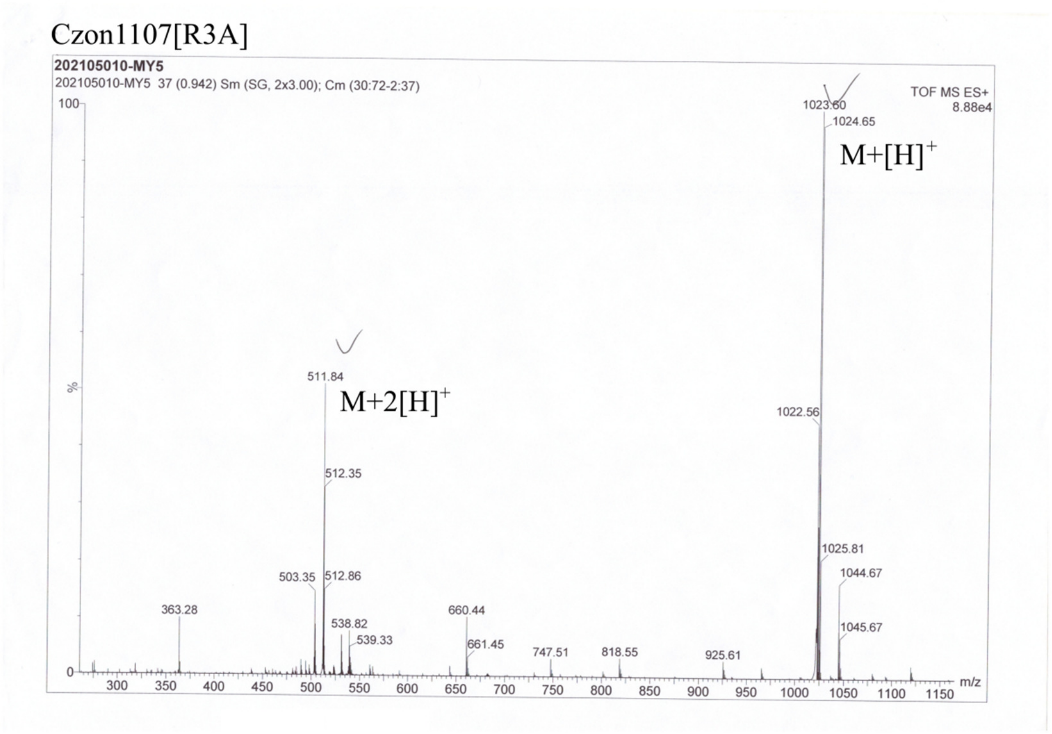

Figure S8. The MS of Czon1107[R3A]

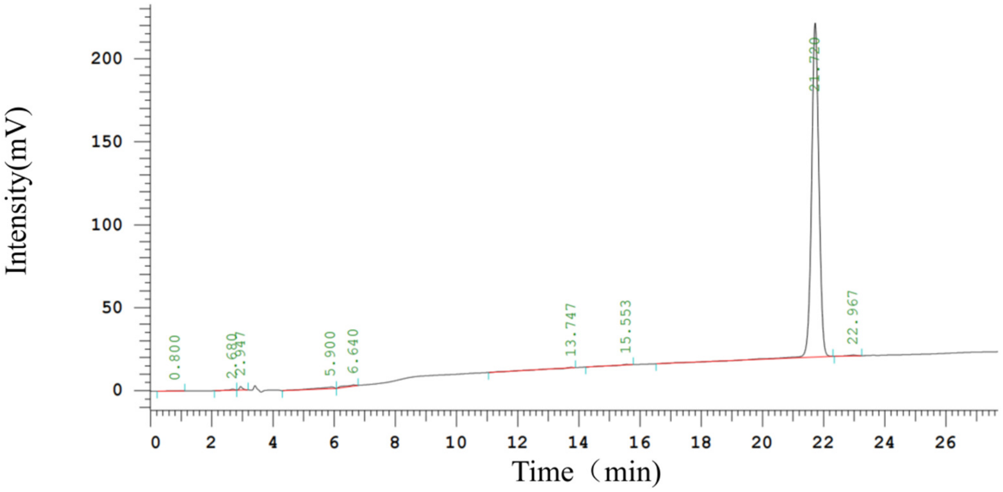

Figure S9. The HPLC of Czon1107[S4A]

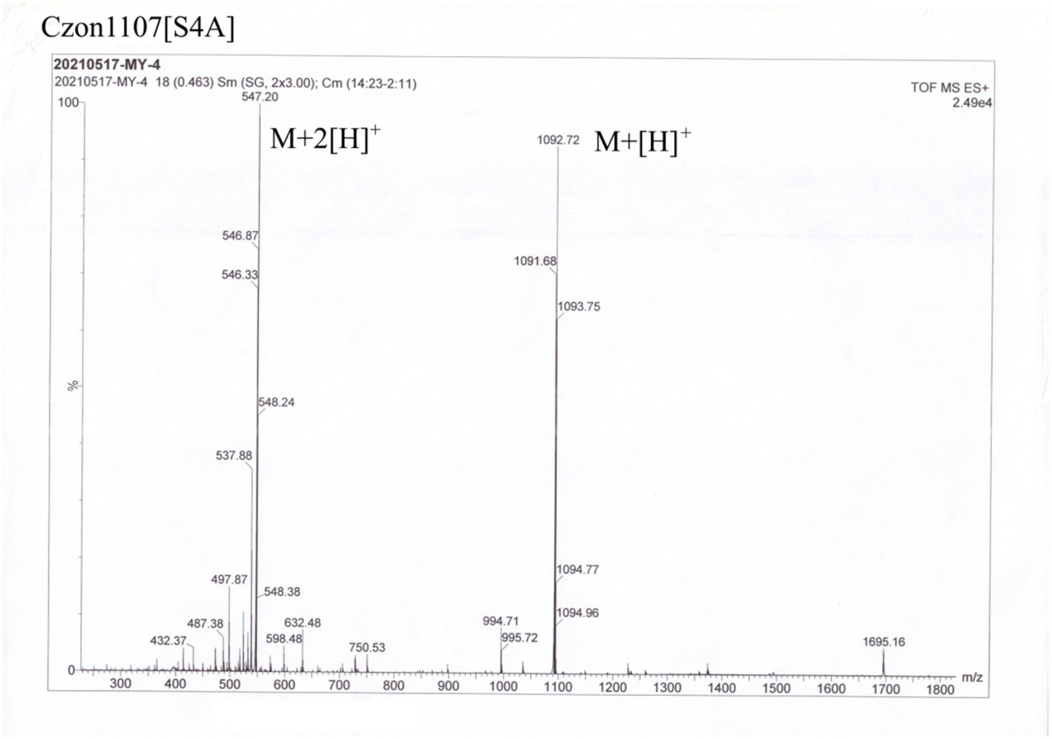

Figure S10. The MS of Czon1107[S4A]

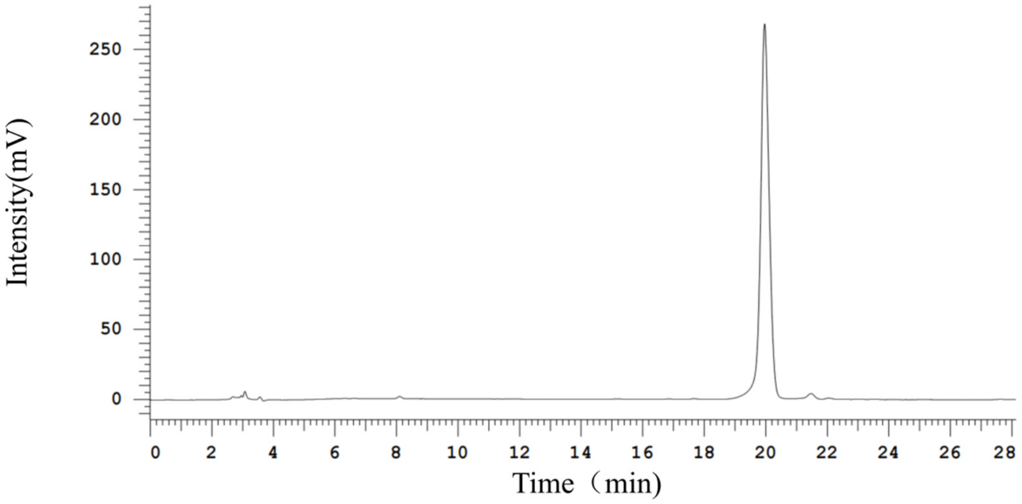

Figure S11. The HPLC of Czon1107[P8A]

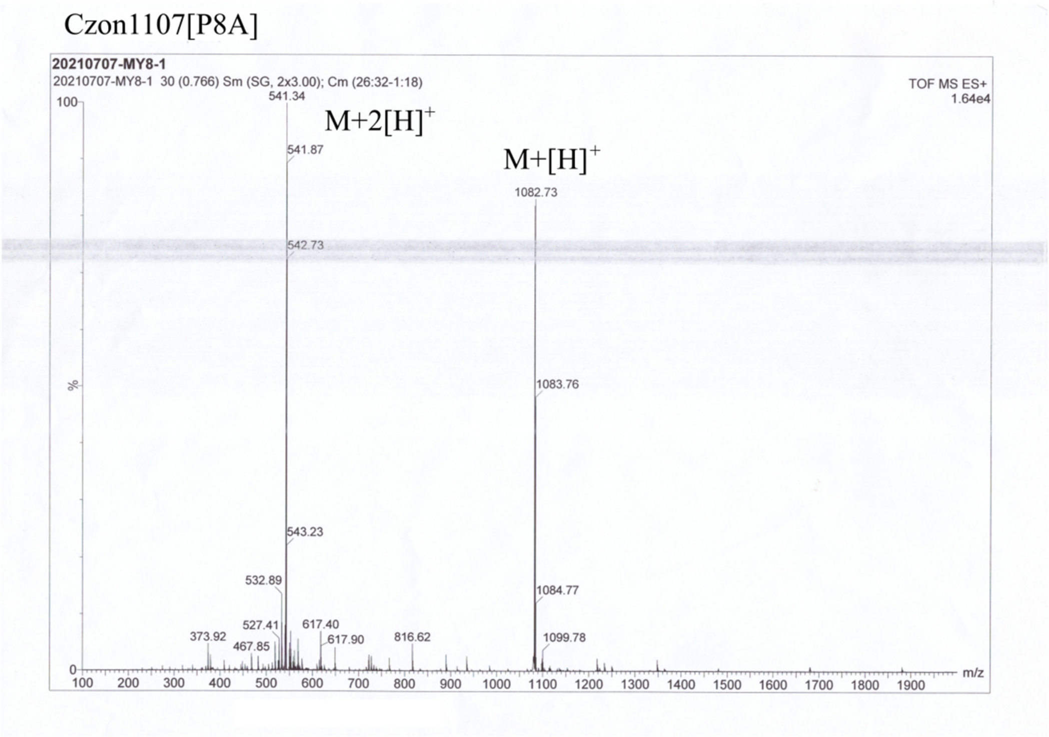

Figure S12. The MS of Czon1107[P8A]

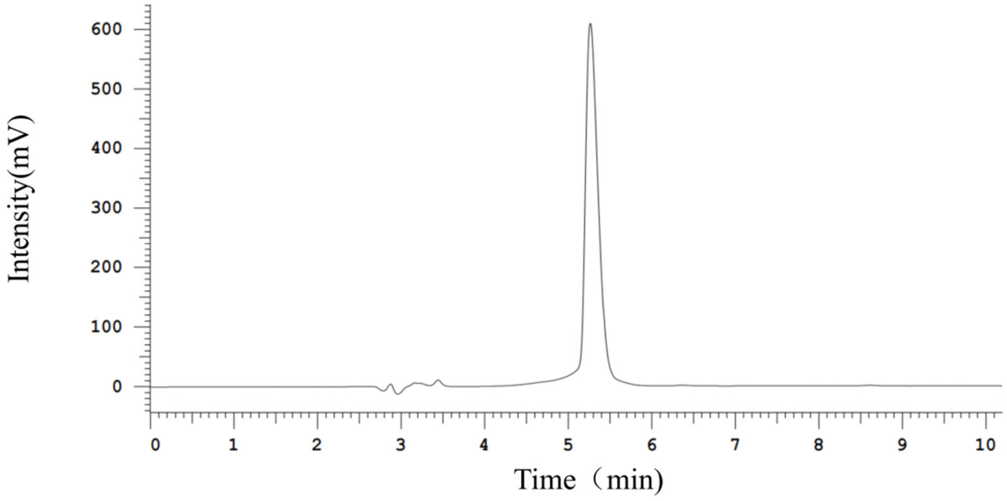

Figure S13. The HPLC of Czon1107[F9A]

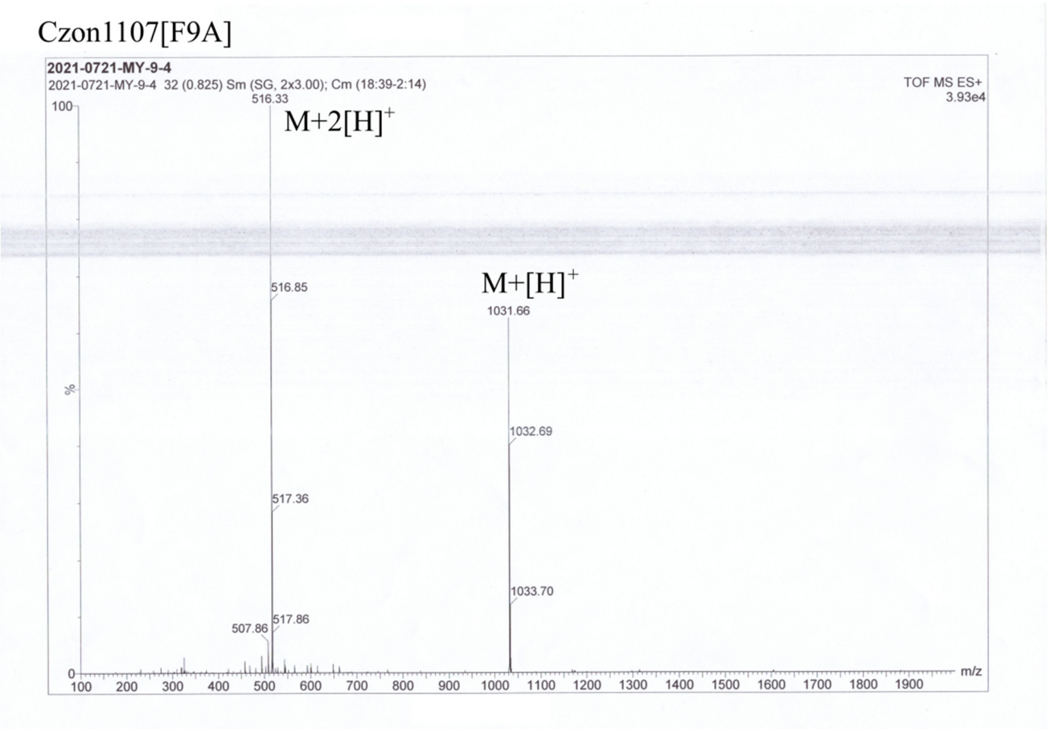

Figure S14. The MS of Czon1107[F9A]

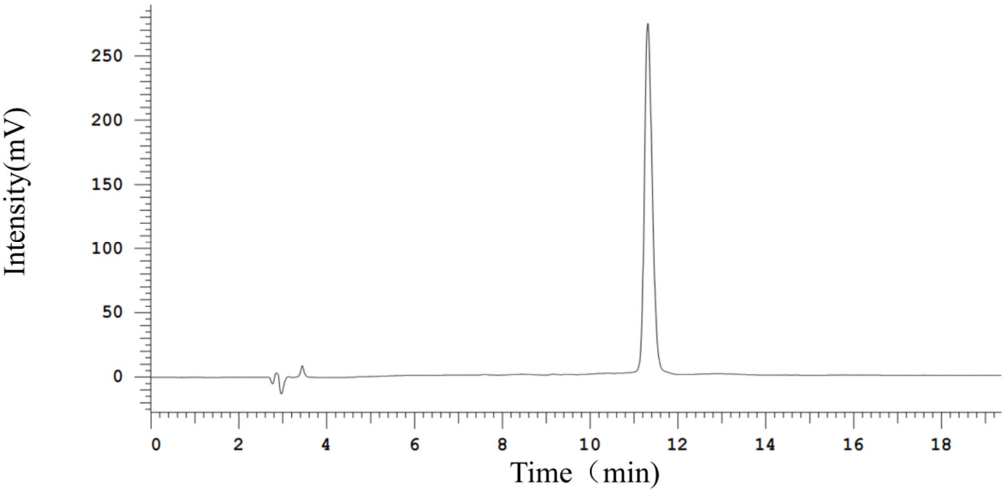

Figure S15. The HPLC of Czon1107[G1R]

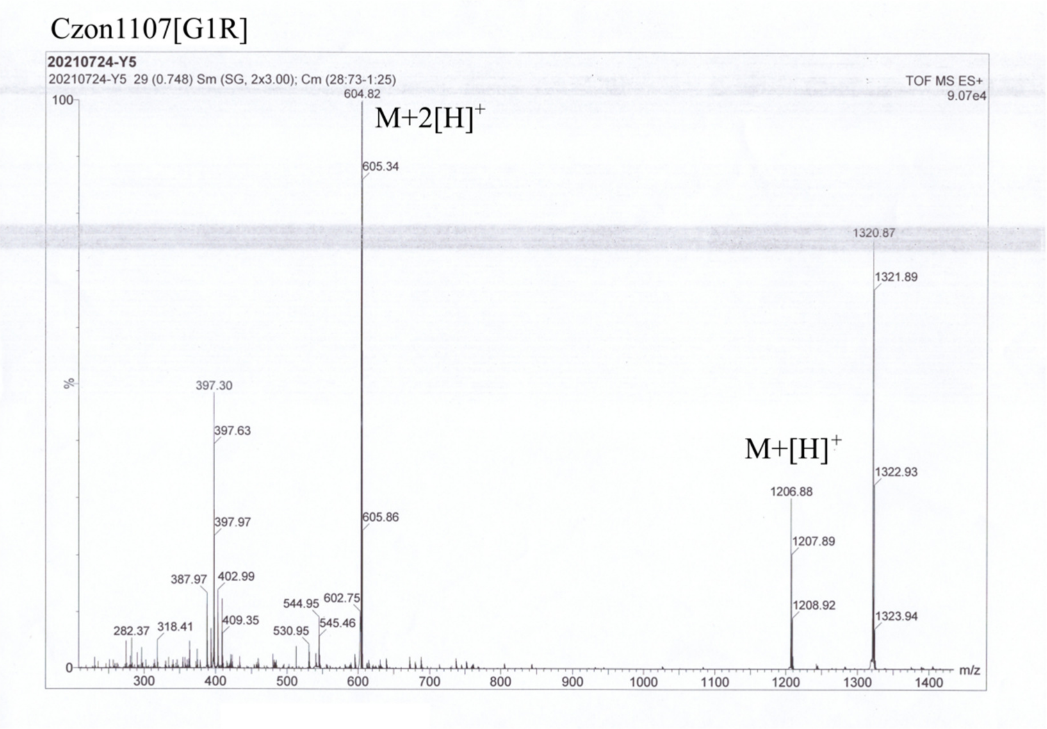

Figure S16. The MS of Czon1107[G1R]

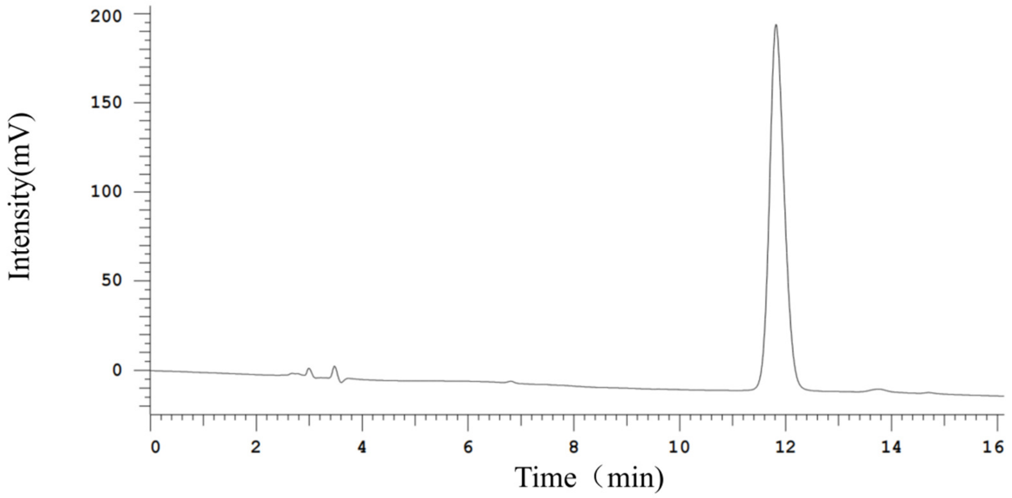

Figure S17. The HPLC of Czon1107[F2R]

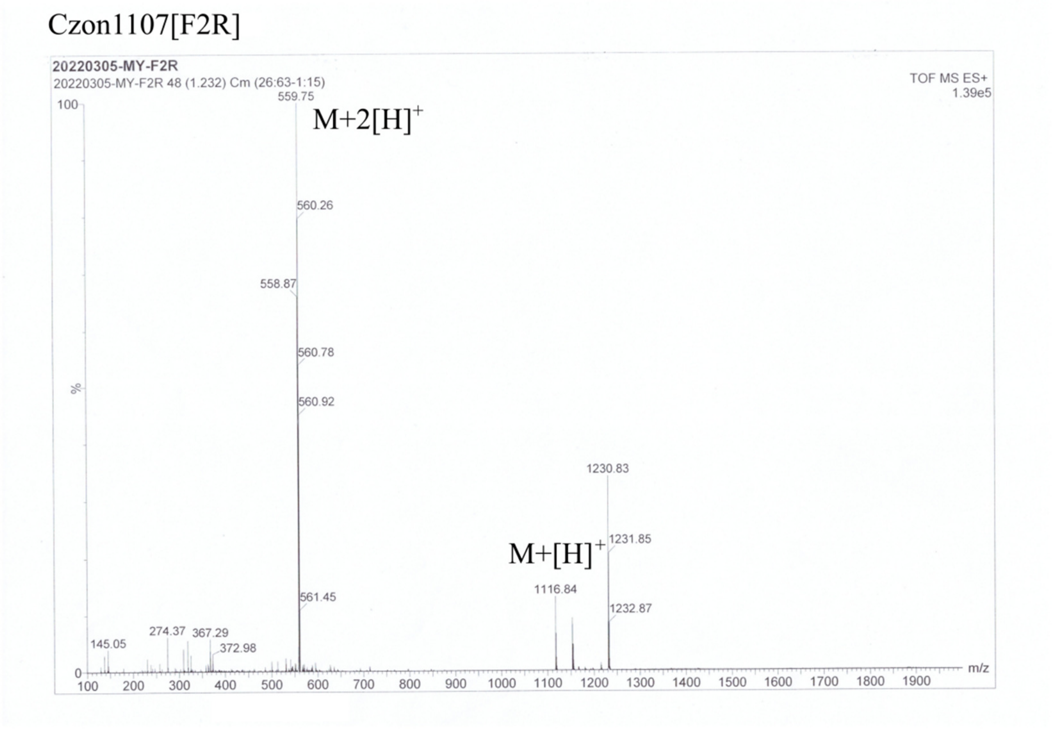

Figure S18. The MS of Czon1107[F2R]

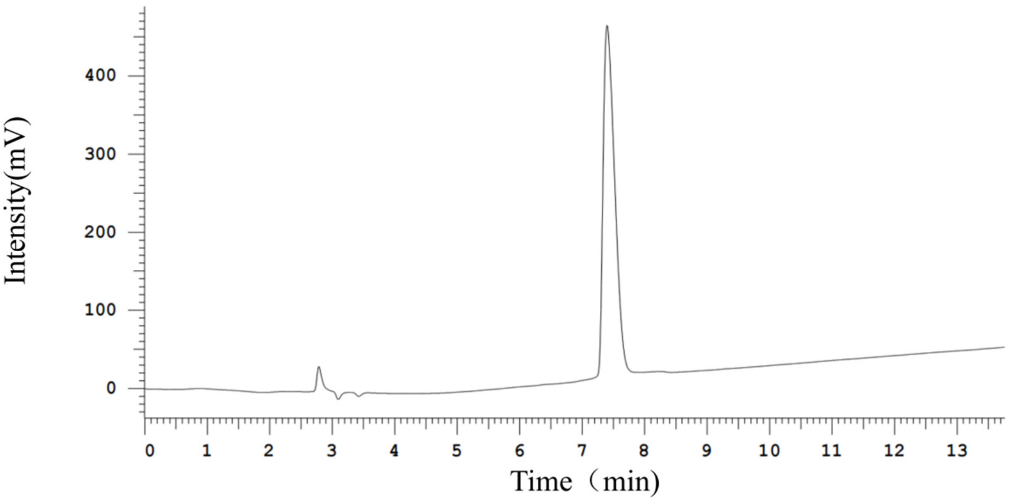

Figure S19. The HPLC of Czon1107[S4R]

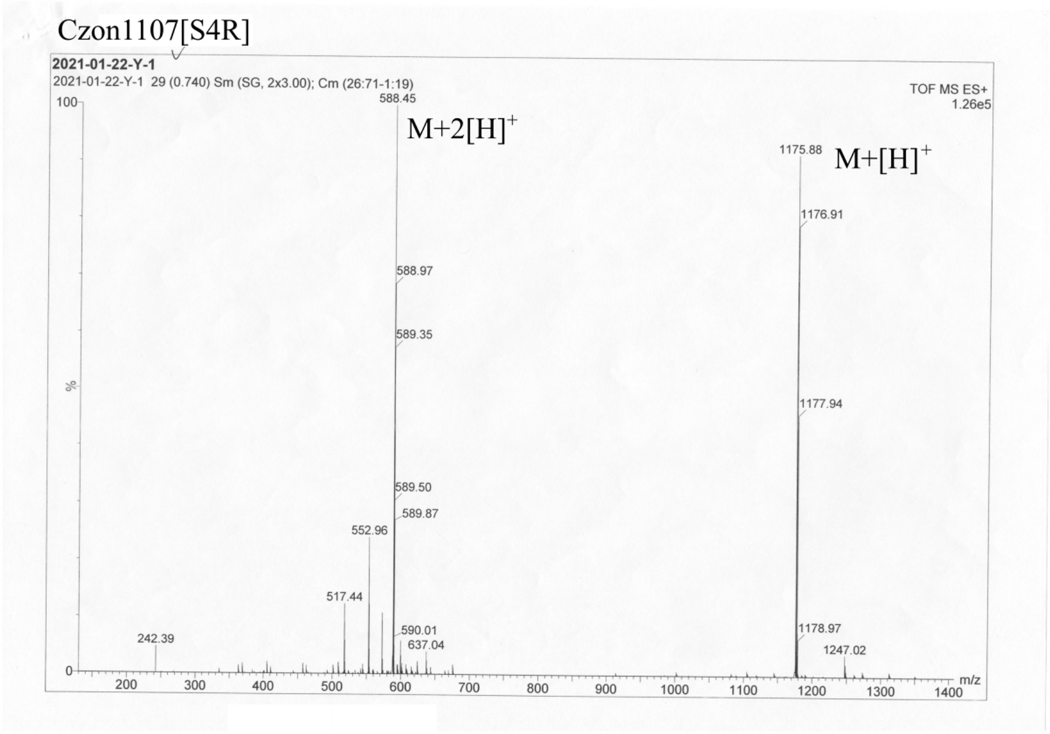

Figure S20. The MS of Czon1107[S4R]

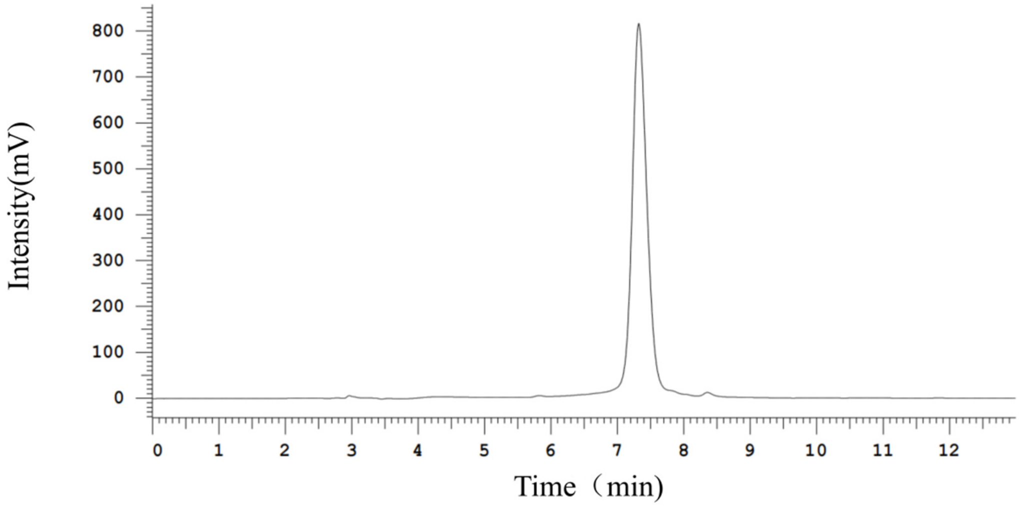

Figure S21. The HPLC of Czon1107[P5R]

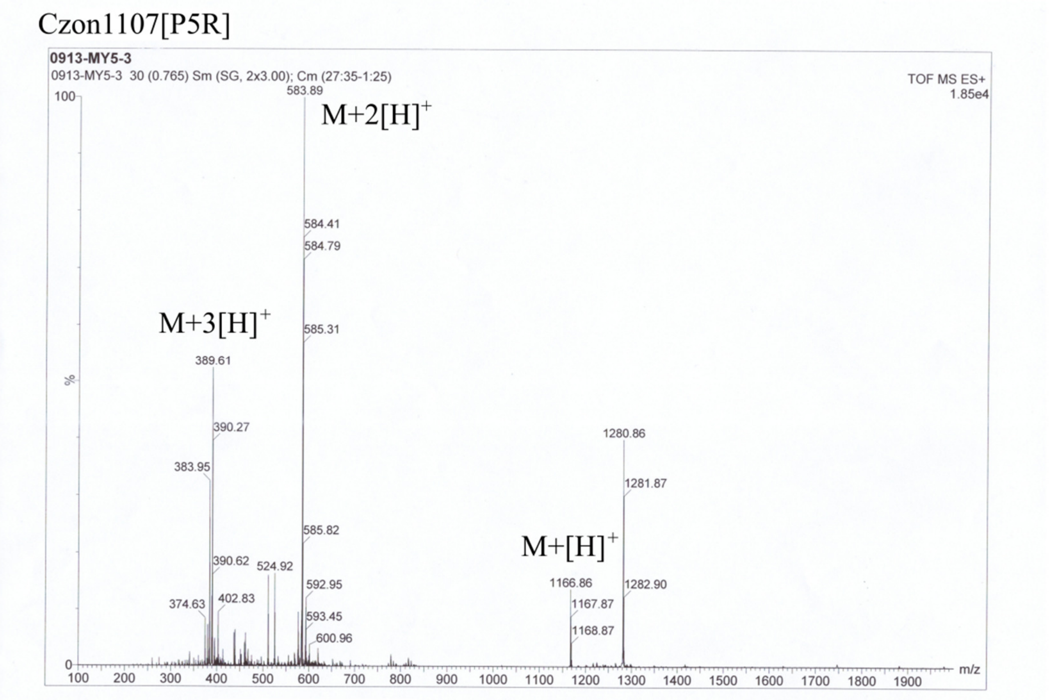

Figure S22. The MS of Czon1107[P5R]

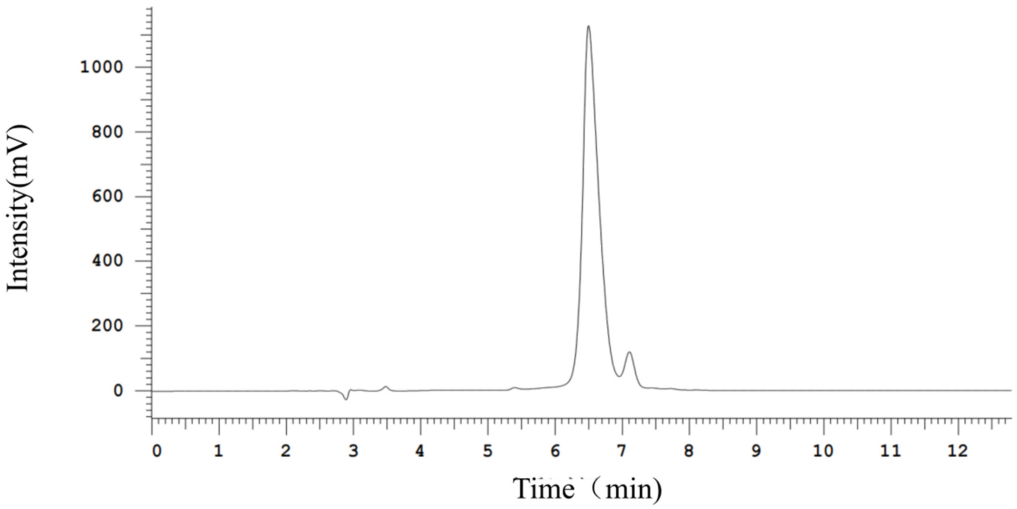

Figure S23. The HPLC of Czon1107[P7R]

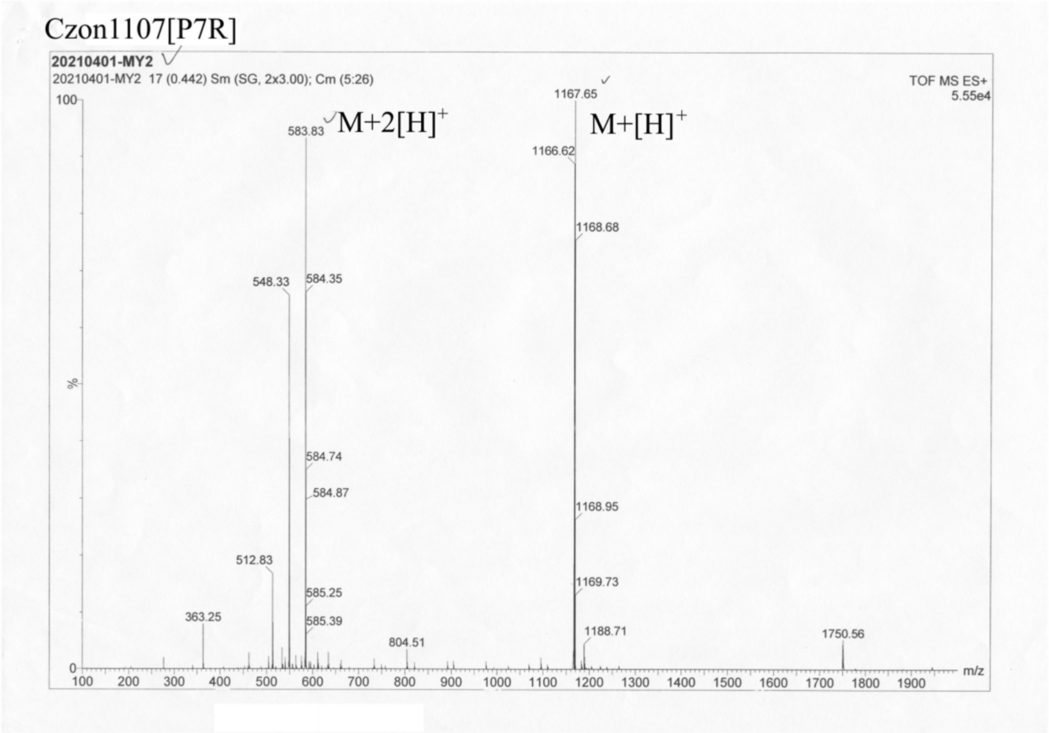

Figure S24. The MS of Czon1107[P7R]

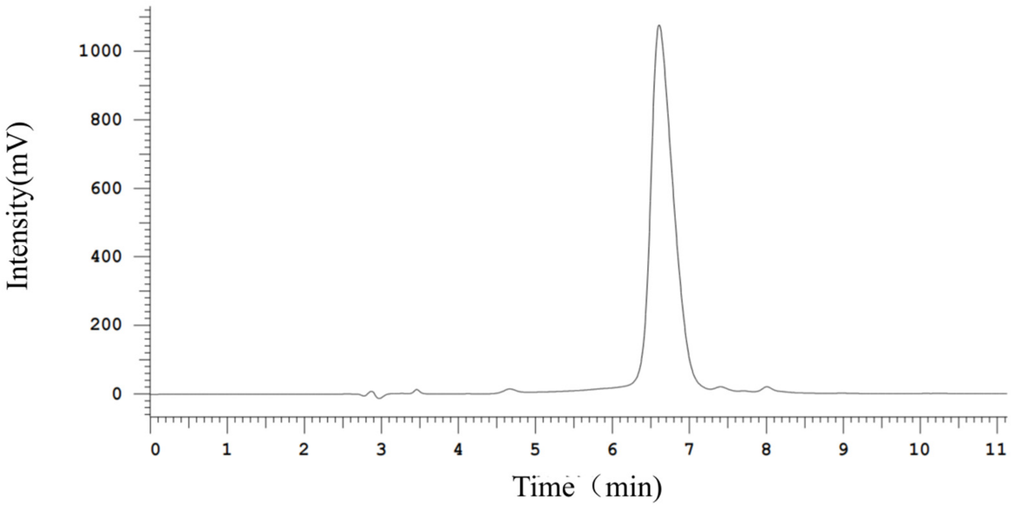

Figure S25. The HPLC of Czon1107[P8R]

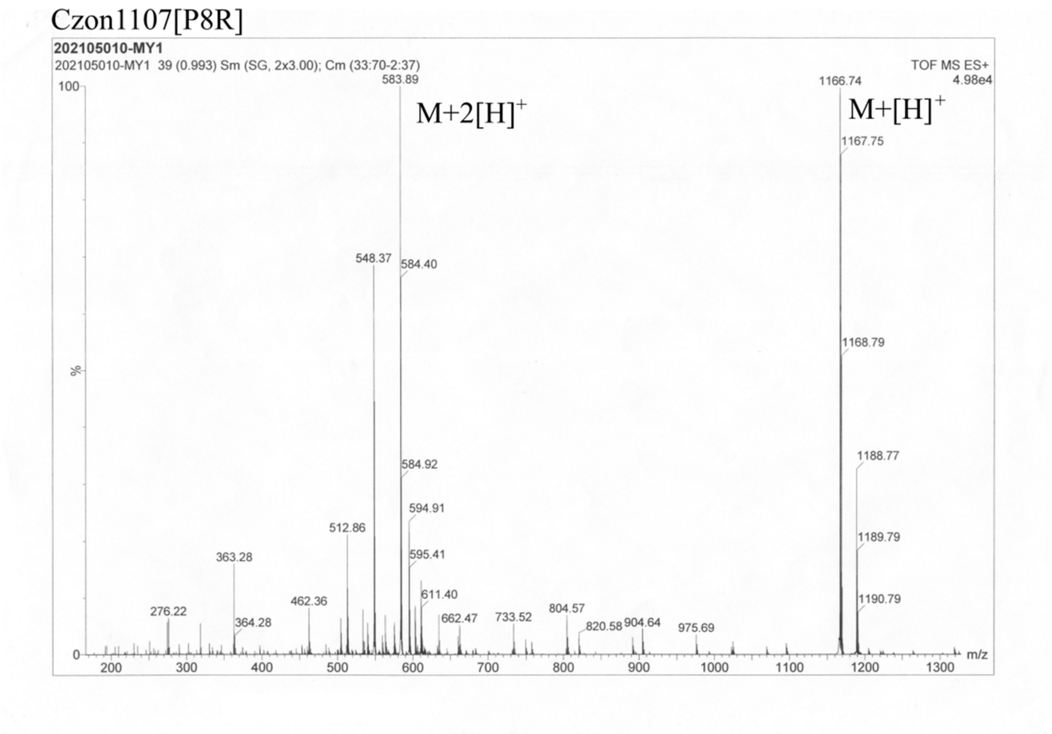

Figure S26. The MS of Czon1107[P8R]

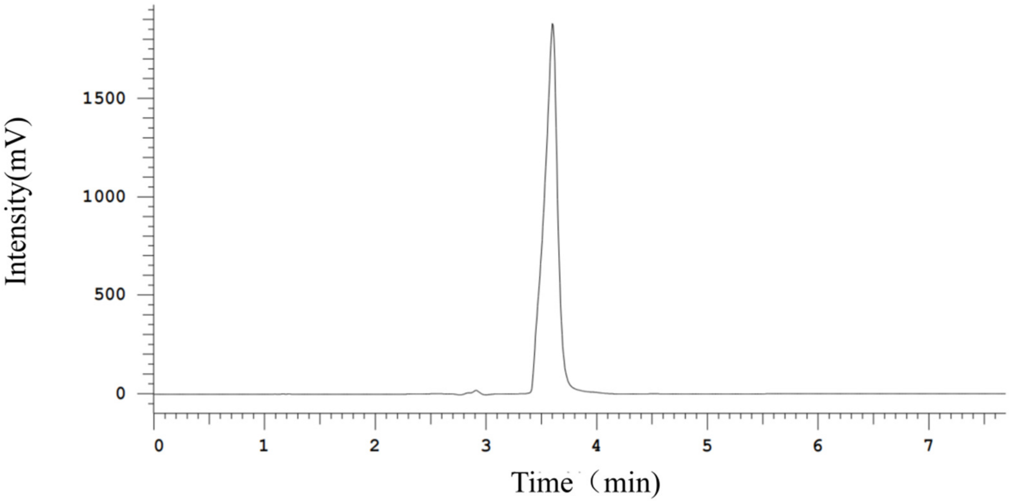

Figure S27. The HPLC of Czon1107[F9R]

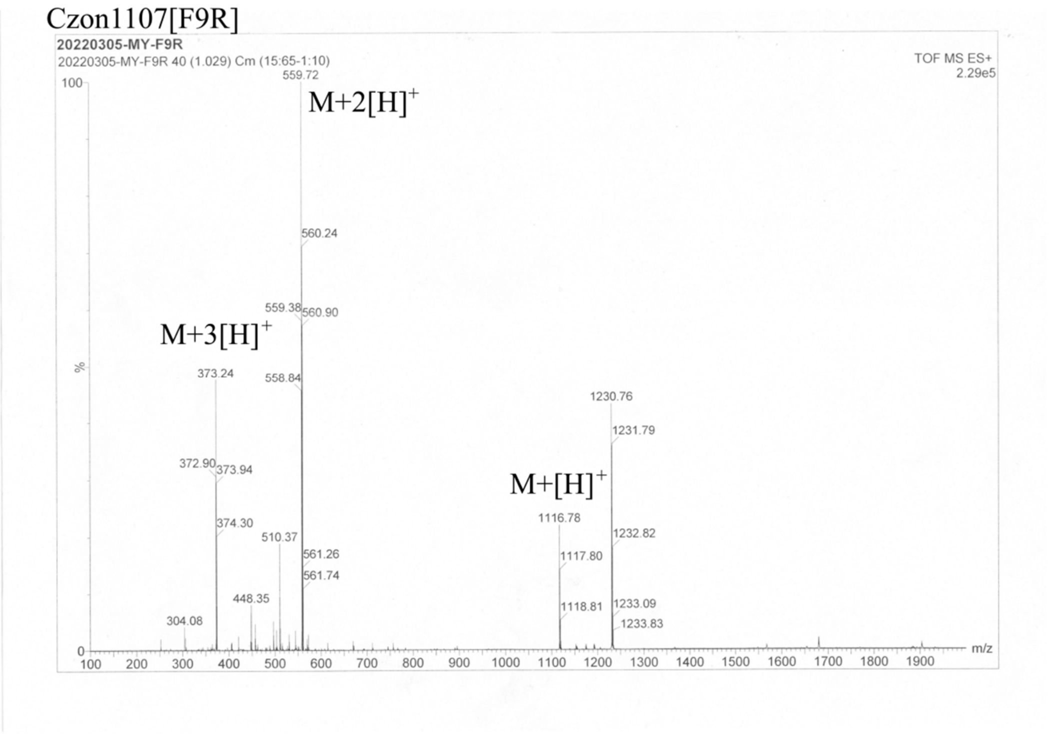

Figure S28. The MS of Czon1107[F9R]

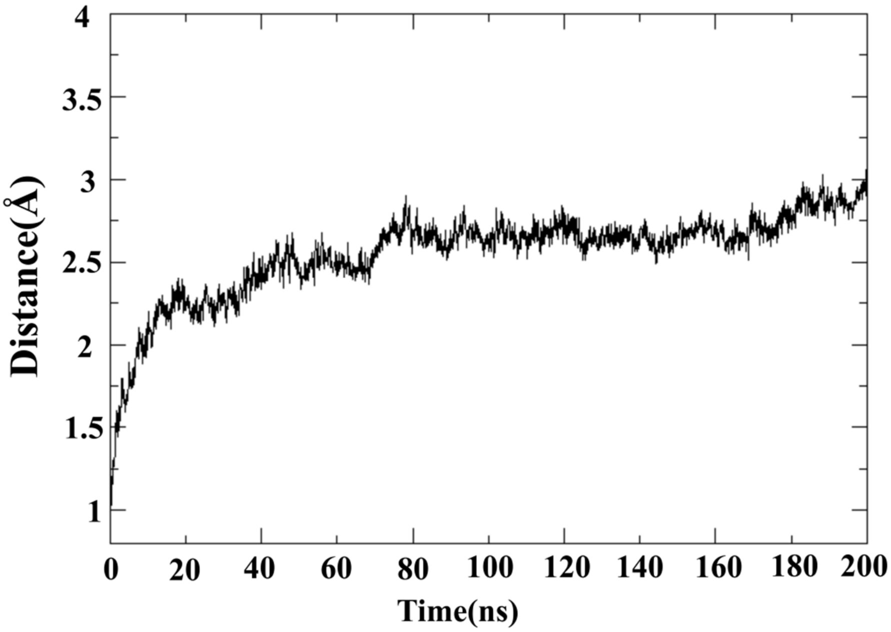

Figure S29. RMSD of Czon1107 bound to hα3β4 nAChR after MD for 200 ns

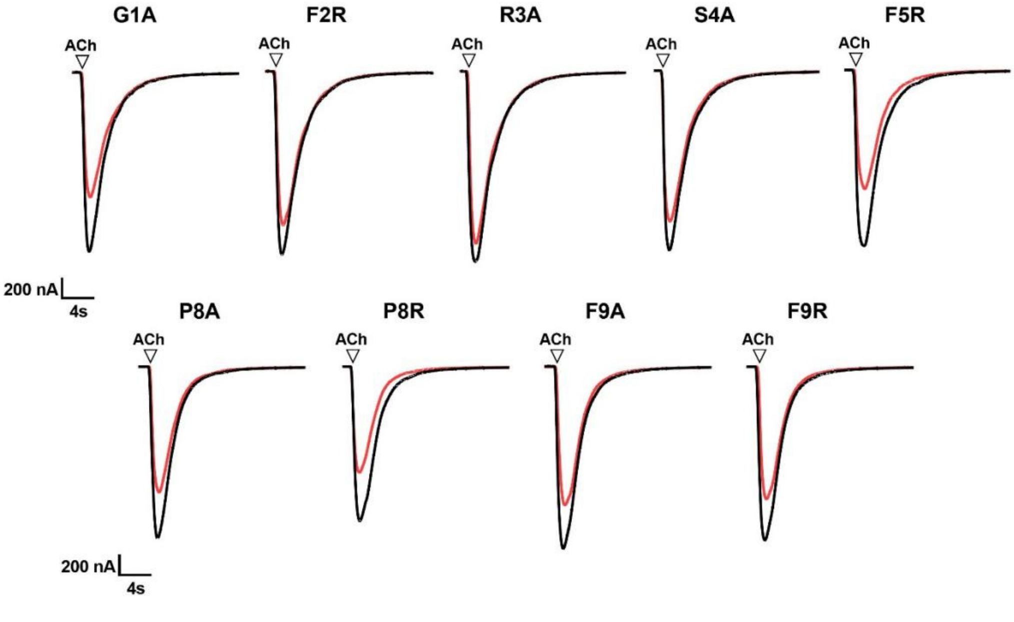

Figure S30. The activity of the labelled Czon1107 analogues (10 μM) on ACh-evoked peak current amplitude mediated by hα3β4 nAChRs. Representative superimposed ACh (300 μM)-evoked currents mediated by hα3β4 nAChRs, obtained in the absence (control, black trace) and presence of 10 μM Czon1107 analogues (red trace).

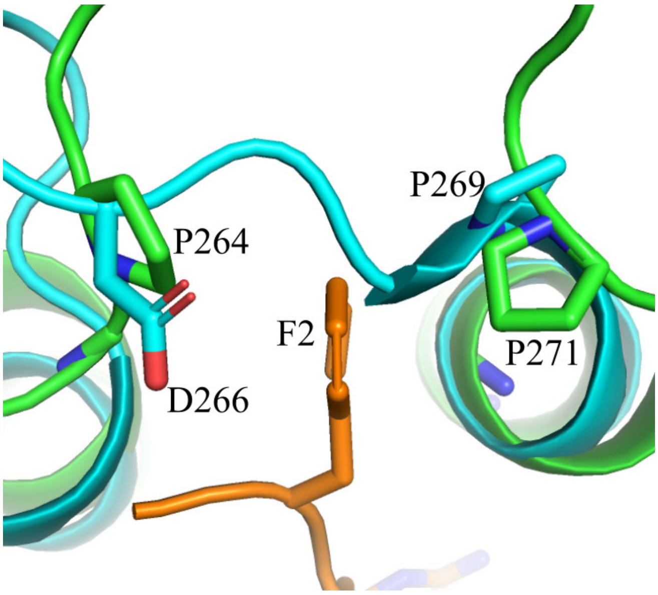

Figure S31. Residue F2 of Czon1107 (orange) interacts with hα3β4 (green) and hα7 nAChRs (cyan). Hydrophobic interactions are involved between Czon1107 residue F2 and hα3β4 residues P264 and P271. At hα7, hydrophobic interactions are involved between Czon1107 residue F2 with hα7 residue P269, but the charge and hydrophilicity of residue D266 affects Czon1107 interactions with hα7 nAChR.
